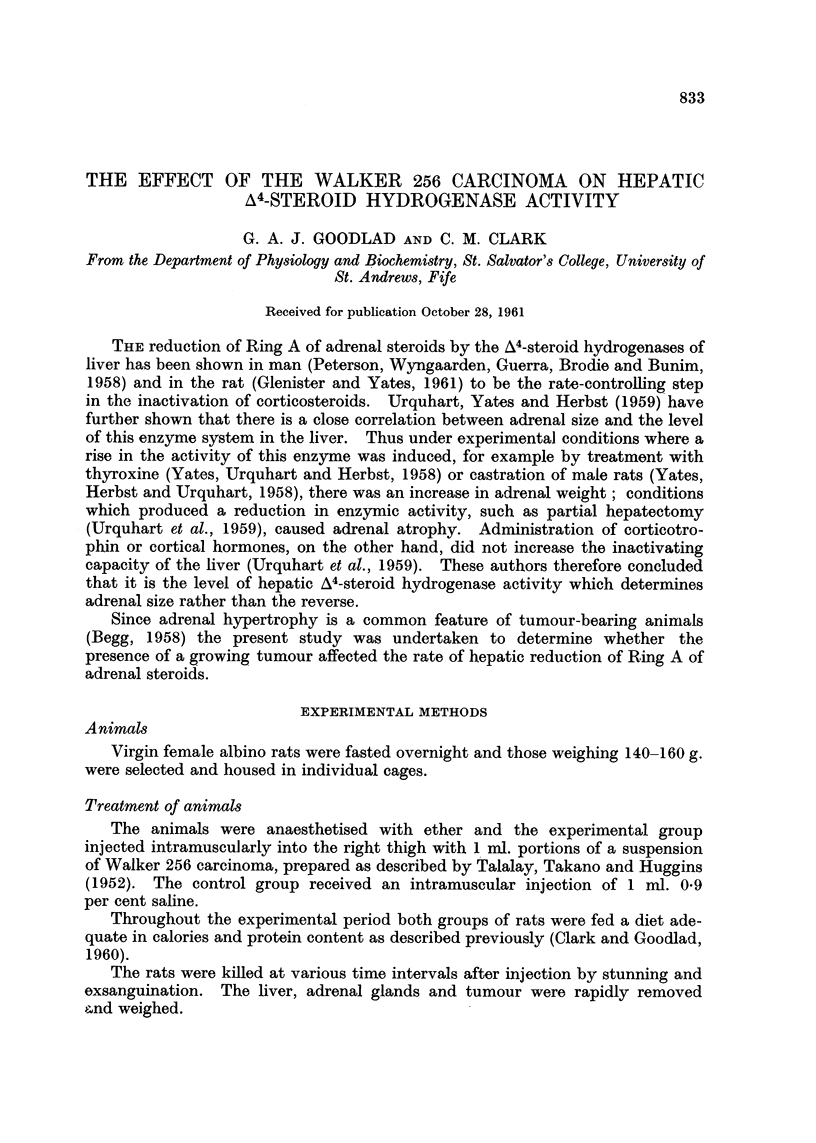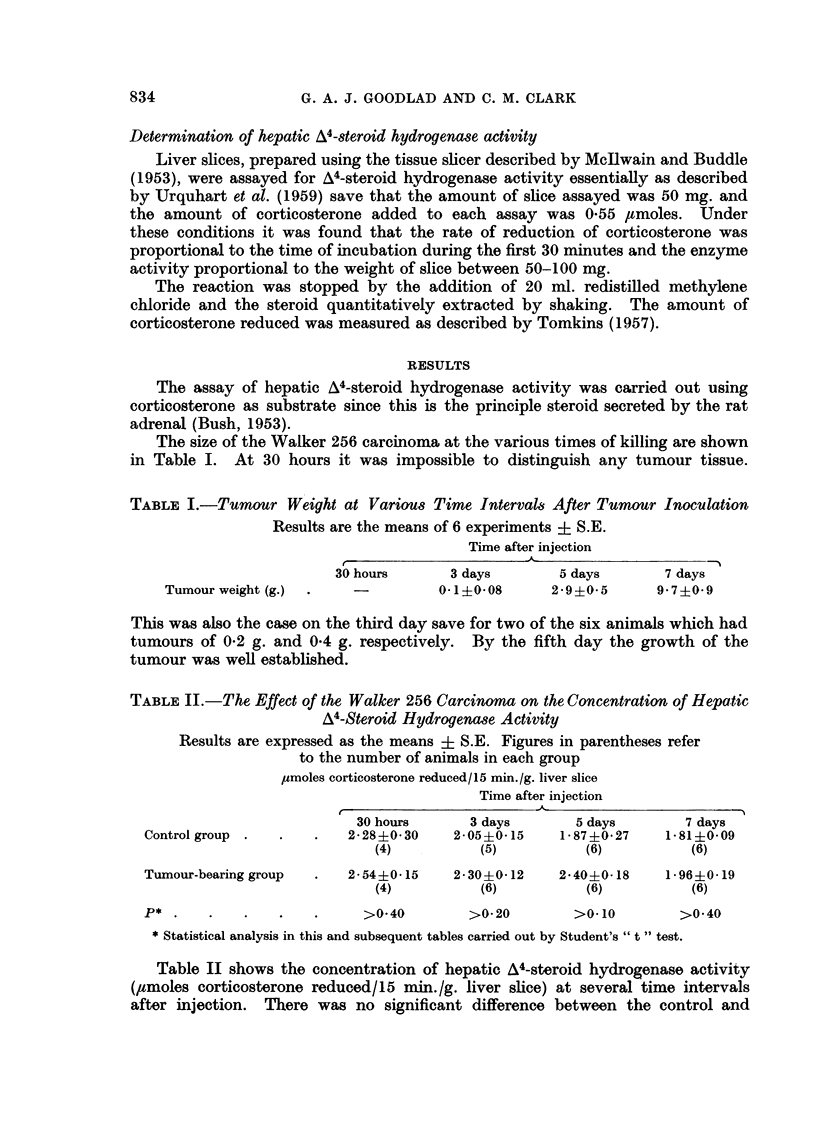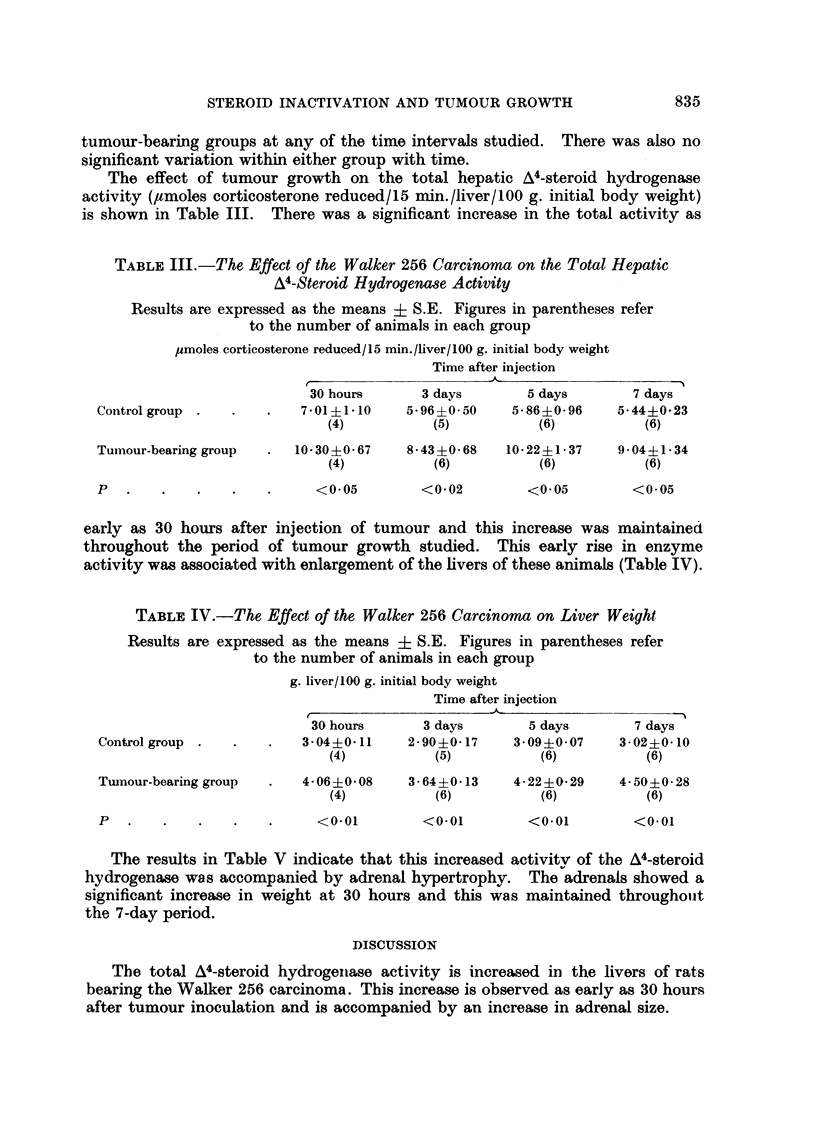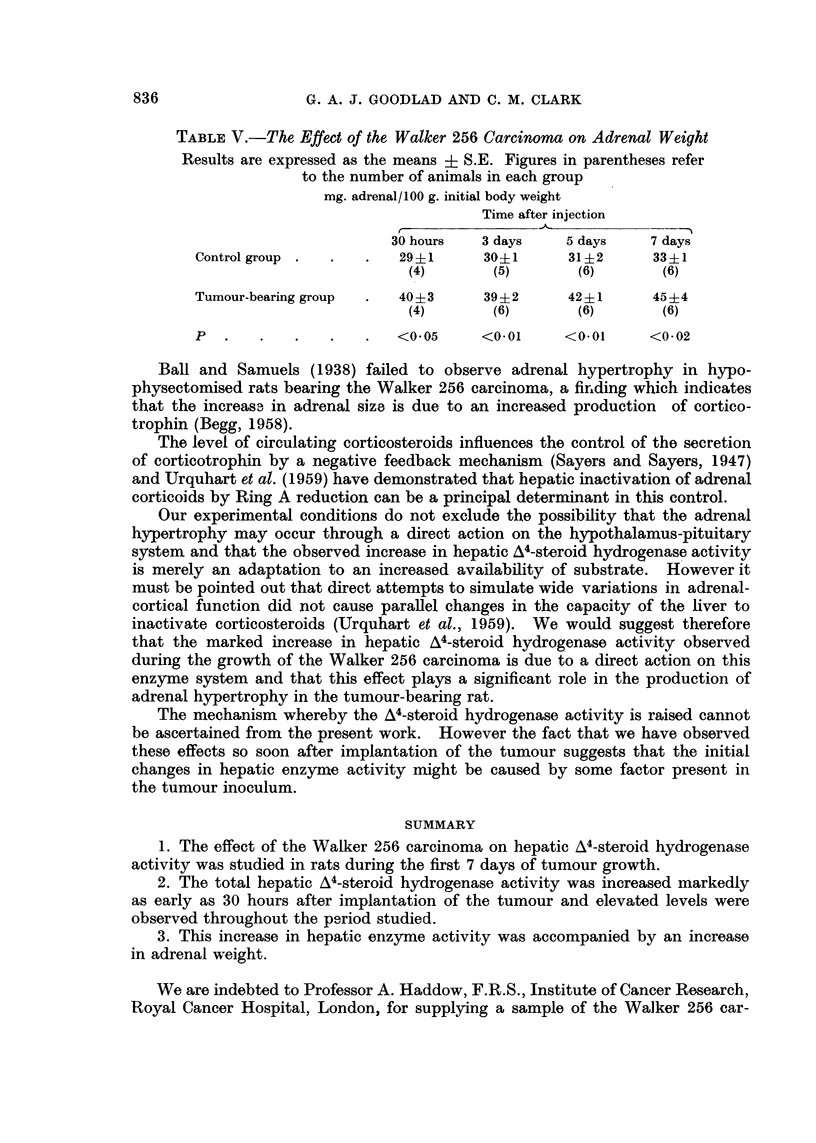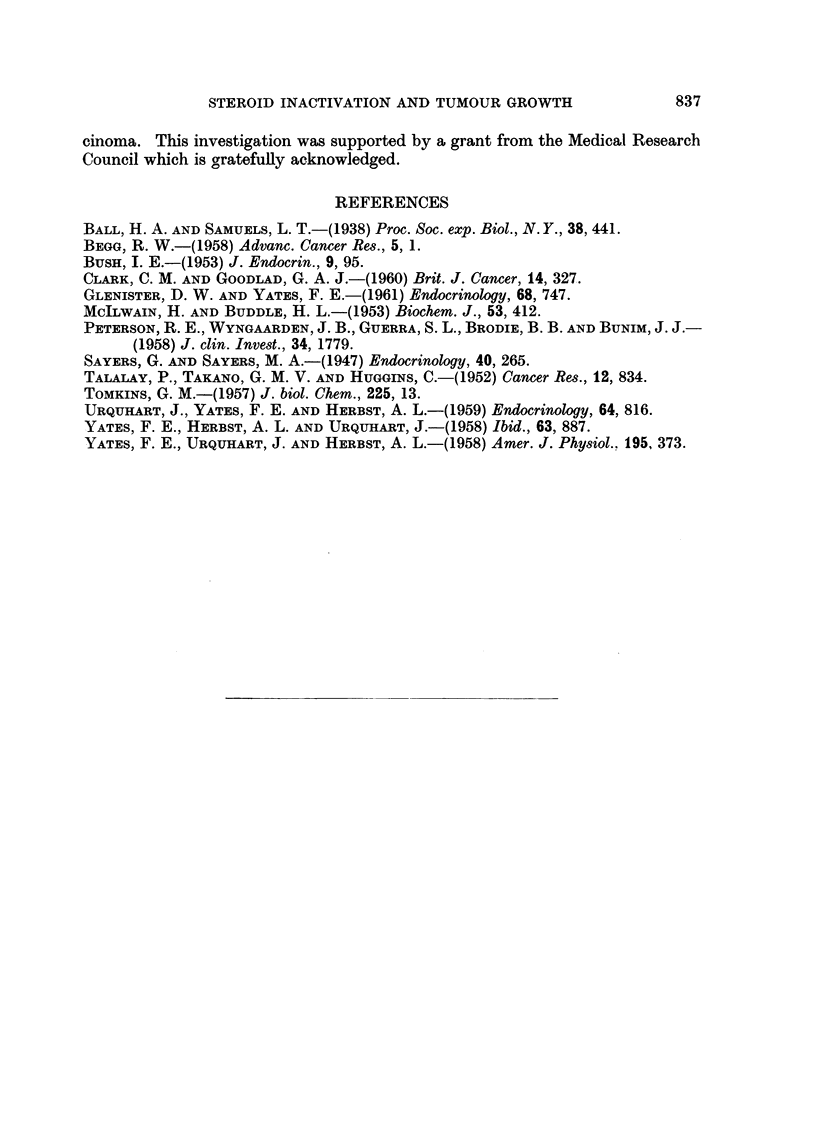# The Effect of the Walker 256 Carcinoma on Hepatic Δ^4^-Steroid Hydrogenase Activity

**DOI:** 10.1038/bjc.1961.96

**Published:** 1961-12

**Authors:** G. A. J. Goodlad, C. M. Clark


### THE EFFECT OF THE WALKER 256 CARCINOMA ON HEPATIC $\Delta^4$-STEROID HYDROGENASE ACTIVITY

G. A. J. GOODLAD AND C. M. CLARK

*From the Department of Physiology and Biochemistry, St. Salvator's College, University of St. Andrews, Fife*

Received for publication October 28, 1961

THE reduction of Ring A of adrenal steroids by the $\Delta^4$-steroid hydrogenases of liver has been shown in man (Peterson, Wyngaarden, Guerra, Brodie and Bunim, 1958) and in the rat (Glenister and Yates, 1961) to be the rate-controlling step in the inactivation of corticosteroids. Urquhart, Yates and Herbst (1959) have further shown that there is a close correlation between adrenal size and the level of this enzyme system in the liver. Thus under experimental conditions where a rise in the activity of this enzyme was induced, for example by treatment with thyroxine (Yates, Urquhart and Herbst, 1958) or castration of male rats (Yates, Herbst and Urquhart, 1958), there was an increase in adrenal weight ; conditions which produced a reduction in enzymic activity, such as partial hepatectomy (Urquhart *et al.*, 1959), caused adrenal atrophy. Administration of corticotrophin or cortical hormones, on the other hand, did not increase the inactivating capacity of the liver (Urquhart *et al.*, 1959). These authors therefore concluded that it is the level of hepatic $\Delta^4$-steroid hydrogenase activity which determines adrenal size rather than the reverse.

Since adrenal hypertrophy is a common feature of tumour-bearing animals (Begg, 1958) the present study was undertaken to determine whether the presence of a growing tumour affected the rate of hepatic reduction of Ring A of adrenal steroids.

### EXPERIMENTAL METHODS

### *Animals*

Virgin female albino rats were fasted overnight and those weighing 140–160 g. were selected and housed in individual cages.

### *Treatment of animals*

The animals were anaesthetised with ether and the experimental group injected intramuscularly into the right thigh with 1 ml. portions of a suspension of Walker 256 carcinoma, prepared as described by Talalay, Takano and Huggins (1952). The control group received an intramuscular injection of 1 ml. 0·9 per cent saline.

Throughout the experimental period both groups of rats were fed a diet adequate in calories and protein content as described previously (Clark and Goodlad, 1960).

The rats were killed at various time intervals after injection by stunning and exsanguination. The liver, adrenal glands and tumour were rapidly removed and weighed.

*Determination of hepatic $\Delta^4$-steroid hydrogenase activity*

Liver slices, prepared using the tissue slicer described by McIlwain and Buddle (1953), were assayed for $\Delta^4$-steroid hydrogenase activity essentially as described by Urquhart *et al.* (1959) save that the amount of slice assayed was 50 mg. and the amount of corticosterone added to each assay was 0·55 $\mu$moles. Under these conditions it was found that the rate of reduction of corticosterone was proportional to the time of incubation during the first 30 minutes and the enzyme activity proportional to the weight of slice between 50–100 mg.

The reaction was stopped by the addition of 20 ml. redistilled methylene chloride and the steroid quantitatively extracted by shaking. The amount of corticosterone reduced was measured as described by Tomkins (1957).

### RESULTS

The assay of hepatic $\Delta^4$-steroid hydrogenase activity was carried out using corticosterone as substrate since this is the principle steroid secreted by the rat adrenal (Bush, 1953).

The size of the Walker 256 carcinoma at the various times of killing are shown in Table I. At 30 hours it was impossible to distinguish any tumour tissue.

TABLE I.—*Tumour Weight at Various Time Intervals After Tumour Inoculation*

Results are the means of 6 experiments ± S.E.

| | Time after injection | | | |
|---|---|---|---|---|
| | 30 hours | 3 days | 5 days | 7 days |
| Tumour weight (g.) . | — | 0·1±0·08 | 2·9±0·5 | 9·7±0·9 |

This was also the case on the third day save for two of the six animals which had tumours of 0·2 g. and 0·4 g. respectively. By the fifth day the growth of the tumour was well established.

TABLE II.—*The Effect of the Walker 256 Carcinoma on the Concentration of Hepatic $\Delta^4$-Steroid Hydrogenase Activity*

Results are expressed as the means ± S.E. Figures in parentheses refer to the number of animals in each group

$\mu$moles corticosterone reduced/15 min./g. liver slice

| | Time after injection | | | |
|---|---|---|---|---|
| | 30 hours | 3 days | 5 days | 7 days |
| Control group . . . | 2·28±0·30 (4) | 2·05±0·15 (5) | 1·87±0·27 (6) | 1·81±0·09 (6) |
| Tumour-bearing group . | 2·54±0·15 (4) | 2·30±0·12 (6) | 2·40±0·18 (6) | 1·96±0·19 (6) |
| *P** . . . . . . | >0·40 | >0·20 | >0·10 | >0·40 |

* Statistical analysis in this and subsequent tables carried out by Student's " t " test.

Table II shows the concentration of hepatic $\Delta^4$-steroid hydrogenase activity ($\mu$moles corticosterone reduced/15 min./g. liver slice) at several time intervals after injection. There was no significant difference between the control and tumour-bearing groups at any of the time intervals studied. There was also no significant variation within either group with time.

The effect of tumour growth on the total hepatic $\Delta^4$-steroid hydrogenase activity ($\mu$moles corticosterone reduced/15 min./liver/100 g. initial body weight) is shown in Table III. There was a significant increase in the total activity as

TABLE III.—*The Effect of the Walker 256 Carcinoma on the Total Hepatic $\Delta^4$-Steroid Hydrogenase Activity*

Results are expressed as the means $\pm$ S.E. Figures in parentheses refer to the number of animals in each group

$\mu$moles corticosterone reduced/15 min./liver/100 g. initial body weight

| | Time after injection | | | |
|---|---|---|---|---|
| | 30 hours | 3 days | 5 days | 7 days |
| Control group | 7·01±1·10 (4) | 5·96±0·50 (5) | 5·86±0·96 (6) | 5·44±0·23 (6) |
| Tumour-bearing group | 10·30±0·67 (4) | 8·43±0·68 (6) | 10·22±1·37 (6) | 9·04±1·34 (6) |
| *P* | <0·05 | <0·02 | <0·05 | <0·05 |

early as 30 hours after injection of tumour and this increase was maintained throughout the period of tumour growth studied. This early rise in enzyme activity was associated with enlargement of the livers of these animals (Table IV).

TABLE IV.—*The Effect of the Walker 256 Carcinoma on Liver Weight*

Results are expressed as the means $\pm$ S.E. Figures in parentheses refer to the number of animals in each group g. liver/100 g. initial body weight

| | Time after injection | | | |
|---|---|---|---|---|
| | 30 hours | 3 days | 5 days | 7 days |
| Control group | 3·04±0·11 (4) | 2·90±0·17 (5) | 3·09±0·07 (6) | 3·02±0·10 (6) |
| Tumour-bearing group | 4·06±0·08 (4) | 3·64±0·13 (6) | 4·22±0·29 (6) | 4·50±0·28 (6) |
| *P* | <0·01 | <0·01 | <0·01 | <0·01 |

The results in Table V indicate that this increased activity of the $\Delta^4$-steroid hydrogenase was accompanied by adrenal hypertrophy. The adrenals showed a significant increase in weight at 30 hours and this was maintained throughout the 7-day period.

### DISCUSSION

The total $\Delta^4$-steroid hydrogenase activity is increased in the livers of rats bearing the Walker 256 carcinoma. This increase is observed as early as 30 hours after tumour inoculation and is accompanied by an increase in adrenal size.

TABLE V.—*The Effect of the Walker 256 Carcinoma on Adrenal Weight*

Results are expressed as the means ± S.E. Figures in parentheses refer to the number of animals in each group mg. adrenal/100 g. initial body weight

| | Time after injection | | | |
|---|---|---|---|---|
| | 30 hours | 3 days | 5 days | 7 days |
| Control group . . . | 29±1 (4) | 30±1 (5) | 31±2 (6) | 33±1 (6) |
| Tumour-bearing group . | 40±3 (4) | 39±2 (6) | 42±1 (6) | 45±4 (6) |
| *P* . . . . . . | <0·05 | <0·01 | <0·01 | <0·02 |

Ball and Samuels (1938) failed to observe adrenal hypertrophy in hypophysectomised rats bearing the Walker 256 carcinoma, a finding which indicates that the increase in adrenal size is due to an increased production of corticotrophin (Begg, 1958).

The level of circulating corticosteroids influences the control of the secretion of corticotrophin by a negative feedback mechanism (Sayers and Sayers, 1947) and Urquhart *et al.* (1959) have demonstrated that hepatic inactivation of adrenal corticoids by Ring A reduction can be a principal determinant in this control.

Our experimental conditions do not exclude the possibility that the adrenal hypertrophy may occur through a direct action on the hypothalamus-pituitary system and that the observed increase in hepatic $\Delta^4$-steroid hydrogenase activity is merely an adaptation to an increased availability of substrate. However it must be pointed out that direct attempts to simulate wide variations in adrenal-cortical function did not cause parallel changes in the capacity of the liver to inactivate corticosteroids (Urquhart *et al.*, 1959). We would suggest therefore that the marked increase in hepatic $\Delta^4$-steroid hydrogenase activity observed during the growth of the Walker 256 carcinoma is due to a direct action on this enzyme system and that this effect plays a significant role in the production of adrenal hypertrophy in the tumour-bearing rat.

The mechanism whereby the $\Delta^4$-steroid hydrogenase activity is raised cannot be ascertained from the present work. However the fact that we have observed these effects so soon after implantation of the tumour suggests that the initial changes in hepatic enzyme activity might be caused by some factor present in the tumour inoculum.

### SUMMARY

1. The effect of the Walker 256 carcinoma on hepatic $\Delta^4$-steroid hydrogenase activity was studied in rats during the first 7 days of tumour growth.

2. The total hepatic $\Delta^4$-steroid hydrogenase activity was increased markedly as early as 30 hours after implantation of the tumour and elevated levels were observed throughout the period studied.

3. This increase in hepatic enzyme activity was accompanied by an increase in adrenal weight.

We are indebted to Professor A. Haddow, F.R.S., Institute of Cancer Research, Royal Cancer Hospital, London, for supplying a sample of the Walker 256 carcinoma. This investigation was supported by a grant from the Medical Research Council which is gratefully acknowledged.

### REFERENCES

BALL, H. A. AND SAMUELS, L. T.—(1938) *Proc. Soc. exp. Biol., N.Y.*, **38**, 441.
BEGG, R. W.—(1958) *Advanc. Cancer Res.*, **5**, 1.
BUSH, I. E.—(1953) *J. Endocrin.*, **9**, 95.
CLARK, C. M. AND GOODLAD, G. A. J.—(1960) *Brit. J. Cancer*, **14**, 327.
GLENISTER, D. W. AND YATES, F. E.—(1961) *Endocrinology*, **68**, 747.
MCILWAIN, H. AND BUDDLE, H. L.—(1953) *Biochem. J.*, **53**, 412.
PETERSON, R. E., WYNGAARDEN, J. B., GUERRA, S. L., BRODIE, B. B. AND BUNIM, J. J.—(1958) *J. clin. Invest.*, **34**, 1779.
SAYERS, G. AND SAYERS, M. A.—(1947) *Endocrinology*, **40**, 265.
TALALAY, P., TAKANO, G. M. V. AND HUGGINS, C.—(1952) *Cancer Res.*, **12**, 834.
TOMKINS, G. M.—(1957) *J. biol. Chem.*, **225**, 13.
URQUHART, J., YATES, F. E. AND HERBST, A. L.—(1959) *Endocrinology*, **64**, 816.
YATES, F. E., HERBST, A. L. AND URQUHART, J.—(1958) *Ibid.*, **63**, 887.
YATES, F. E., URQUHART, J. AND HERBST, A. L.—(1958) *Amer. J. Physiol.*, **195**, 373.